# The Cost of Prospective Memory in Children: The Role of Cue Focality

**DOI:** 10.3389/fpsyg.2018.02738

**Published:** 2019-01-09

**Authors:** Ana B. Cejudo, Carlos J. Gómez-Ariza, M. Teresa Bajo

**Affiliations:** ^1^Department of Experimental Psychology, The Mind, Brain, and Behaviour Research Centre, University of Granada, Granada, Spain; ^2^Department of Psychology, University of Jaén, Jaén, Spain

**Keywords:** prospective memory, cue focality, cognitive cost, children, development

## Abstract

Prospective memory (PM) is an essential ability in daily life, since it involves remembering to perform an intention. While PM largely develops during childhood and adolescence, its underlying mechanisms are still poorly understood. In general, age differences in PM have been found with tasks in which the prospective cues are not part of the ongoing activity (non-focal PM tasks). In the present study, we evaluated the cognitive cost produced by a PM task over the ongoing activity by comparing the performance of a single-task condition with that of an ongoing activity condition involving a prospective intention. Specifically, to determine the impact of cue focality on performance as a function of age, we tested two groups of children (6 and 11 years old) in three experimental conditions: single, focal and non-focal prospective cues. In the single-task condition, children were only asked to perform the ongoing task (to categorize images as animal or non-animal). In the focal condition, in addition to performing the ongoing activity, participants were asked to press different keys whenever the image appearing on the screen was a kite or a ball. In the non-focal condition, children were to press the keys if the color of the frame of the screen changed to magenta or gray. Although reaction times were greater for the non-focal conditions in both age groups, the results showed worse performance on the ongoing activity for both the focal and the non-focal conditions (relative to the single-task condition) in the younger children. This difference was less pronounced in older children so that response times for focal and non-focal cues differed from the single condition, but the difference in performance between focal and single task conditions was not reliable. These findings, which are partly in line with the dual process framework (McDaniel et al., 2015), suggest that while non-focal prospective cues compromise attentional control in younger and older children, focal cues seem to rely on less effortful processes in older children.

## Introduction

Prospective memory (PM) is the ability to remember to complete a future intention (Brandimonte et al., 1996). This ability is essential to success in daily life activities, such as remembering to make an important call or take a pill after breakfast. In children, low PM performance could disrupt school life; for example, a child may forget to give his/her parents a permission slip or bring his/her homework to class (Kvavilashvili et al., 2001). In a typical PM task, participants are asked to carry out an ongoing task (OT) while also remembering to perform a prospective task, either when they encounter a specific cue embedded within the OT or when a specific time has elapsed (Kvavilashvili et al., 2001). Prospective recall is a time-based PM task that requires the person to remember to perform an action at a specific time or time interval, and event-based PM tasks involve remembering to perform an intention upon the occurrence of a specific event (McDaniel and Einstein, 2000). The present study focuses on the latter type of PM task and tries to identify age differences in the possible costs associated with maintaining a prospective intention while performing an ongoing task.

Previous research has suggested that successfully remembering an intention involves four main processes: forming an intention, maintaining the intention until the appropriate cue or time is present, initiating the intended action when the cue is detected (event or time) and, finally, executing the intention (Kliegel et al., 2002). According to the preparatory attentional and memory processes (PAM) theory, these processes consume attention and generate a cost in the OT (Smith, 2003; Smith et al., 2010). Thus, to monitor the environment for cues that signal retrieval of the intention, participants should maintain a state of readiness during the OT. Although these processes may be outside of conscious awareness, they consume resources, impairing OT performance. This claim has been supported by various experiments reporting slower performance and lower accuracy for the OT while trying to remember an intention, relative to a control condition in which the OT is performed by itself (Craik et al., 1996; Park et al., 1997; Smith, 2003; Anderson et al., 1998; Smith et al., 2007). For example, Smith (2003) reported that participants were 300 milliseconds (ms) slower in performing a lexical-decision task (deciding whether or not a string of letters formed a word) when they were also instructed to prospectively remember a particular word (PM intention) than when they were not asked to remember a word. Similarly, Smith et al. (2010) reported lower performance in a color-matching task when participants were required to press another key when a particular image appeared on the screen (PM task). In general, results comparing OTs with and without concurrent prospective intentions suggest that participants strategically allocate resources to monitor PM cues, imposing a cost on the OT.

Strategic allocation of resources to a PM task has also been related to working memory (WM) capacity. WM is needed to keep an intention in mind and to update the task goal when a cue is encountered (Einstein et al., 2000). Several studies have reported a relationship between WM and prospective recall performance (Smith and Bayen, 2005; Wang et al., 2008; Mahy and Moses, 2011). For example, Smith and Bayen (2005) found that WM capacity predicted the extent to which participants engaged in preparatory attentional processes to perform a PM task. Participants with higher span scores showed greater costs than participants with lower span scores in the OT, indicating that high-span participants were more prone to engage in preparatory attentional processes. Similarly, Cheie et al. (2017) showed that increasing processing demands on the OT or imposing an additional WM span on children compromised their performance.

However, the PAM theory assumption that prospective remembering always requires preparatory attentional processes has been questioned. According to the dual process framework, PM retrieval could be spontaneous or effortful, depending on the task demands (Einstein et al., 1997; McDaniel et al., 2015). For example, Basso et al. (2010) manipulated the cognitive demands of WM and PM dimensions on an event-based prospective task. The ongoing activity was either a WM-updating task involving higher or lower demands or a lexical decision task (low WM demands). The prospective task required the participants to respond whenever a previously presented word appeared. The results pattern was complex because PM only affected performance on the WM task at higher loads. By contrast, the pattern for the lower WM conditions showed that performance was independent of the concurrent PM task. Similarly, a number of studies have shown no cost to the OT with successful PM performance (Harrison and Einstein, 2010; Scullin et al., 2010; Knight et al., 2011; Scullin et al., 2011), suggesting that, in some cases, cue monitoring might not be attentionally costly.

More direct evidence for the dual process framework (McDaniel et al., 2015) comes from studies manipulating the focality of the prospective cue. Focality is manipulated under the assumption that the degree to which attentional resources are demanded for cue monitoring depends on whether the PM task involves focal or non-focal cues. Focal PM tasks are those in which the OT involves processing the defining features of the PM cues (e.g., categorizing strings of letters as words/non-words and pressing another key whenever a predetermined target word appears as a PM task; Einstein and Mcdaniel, 2005). By contrast, non-focal tasks involve PM cues that are not part of the information extracted from the OT for accurate performance (e.g., deciding whether the word on the left is a member of the category on the right as an OT and pressing another key whenever the word includes the syllable “tor”; Einstein and Mcdaniel, 2005). In focal PM tasks, the OT forces processing of the PM target, potentially requiring spontaneous non-attentional retrieval. By contrast, in non-focal PM tasks, monitoring for external cues is necessary because there is no overlap between the information needed for the OT and that needed for PM performance. In this case, effortful monitoring should be invested to detect the PM cue and to switch from the OT to the PM. According to this proposal, cue focality should have clear effects on monitoring and cue detection, since the ability to strategically monitor for environmental cues may depend on whether the OT orients attention to the relevant contextual PM cue. For example, in Ball and Bugg’s (2018, Experiment 1) study, participants were asked to perform a lexical decision task (OT) and to detect a syllable embedded in some words. They were specifically told that the syllable occurred in words, but not in non-word trials (focal context cue condition). By contrast, in the non-focal condition, they were told that the syllable appeared only in items starting with consonants (non-focal context cue condition). Strategic monitoring (resulting in an OT cost for the PM condition relative to the single-OT control condition) was only evident during the focal condition, in which the type of OT processing automatically oriented attention to the relevant features of the contextual cue. These findings suggest that strategic monitoring is dependent on limited-capacity processing resources and may be relatively limited when the attentional demands of context identification are sufficiently high.

Because these processes require efficient WM and executive control capacity, another important factor in PM performance is age. In general, research has shown that the development of PM across the lifespan follows an inverted U path, with PM increasing from preschool to adolescence and decreasing from late adulthood (Zimmermann and Meier, 2006; Zöllig et al., 2007). OT costs have been found in adult populations, but also in children. For example, Leigh and Marcovitch (2014) reported PM costs in young children (4, 5, and 6 years old) categorizing images (as animals/non-animals or food/non-food) when they were also asked to press a smiley face button whenever they saw a particular image. However, although some studies have included OT performance as a covariate (Kliegel and Jäger, 2007; Kvavilashvili et al., 2008), few studies have focused on PM costs during OT performance by themselves, and these effects are not completely understood (Leigh and Marcovitch, 2014). In addition, the lower performance of children in OTs might be due to less efficient cognitive processing (resulting in more costly PM), but also to deficient strategies related to allocating resources to the PM task.

One way to approach this problem is to manipulate the attentional demands of the PM task (e.g., by manipulating cue focality) and explore the effects of this manipulation in children of different ages. This approach has been followed with younger and older adults (see Henry et al., 2004, for a review). For example, Rendell et al. (2007) manipulated the presence of focal and non-focal PM cues in younger and older adults and found that age-related differences on PM performance were more pronounced when the cue was non-focal.

Age differences in more demanding non-focal tasks have also been shown in studies comparing adolescents and young adults (Wang et al., 2011). Wang et al. (2011) manipulated the focality of the cue and found that the adult group outperformed the adolescent group in the non-focal PM condition. They used ongoing spatial WM tasks in which participants were asked to press a target key whenever a specific embedded target appeared (focal condition) or whenever the background color of the WM trials changed to a specific color (non-focal). Response times for the ongoing WM task showed group differences only when the PM task involved non-focal intentions, suggesting that age differences might specially arise in cases involving more difficult monitoring and cue detection. Finally, Kliegel et al. (2013) compared 6- and 7-year-old and 9- and 10-year-old children who played a videogame requiring them to drive a vehicle. In the non-focal condition, the PM cue was a yellow flowerpot located outside the road, and in the focal condition, the cue was a yellow car also in the road. Performance in the PM task was lower in the 6- and 7-year children than the 9- and 10-year-old children in both conditions, suggesting that both focal and non-focal cues require attentional resources. However, when performance on the OT was included as a covariate, age differences appeared when the cue was outside of the center of attention.

However, Kliegel et al. (2013) found mixed results and did not report direct comparisons of the children’s performance on the OT. Thus, the main aim of the present study was to address the role of cue focality in children of different ages by examining PM performance and ongoing cost. We directly compared children’s (6 and 11 years old) performance in the OT in conditions in which they performed the OT by itself (single-task condition) and in conjunction with a focal or a non-focal PM task. We chose 6-year-olds and 11-year-olds for our groups because previous research has shown differences in PM between these two age groups (Smith et al., 2010; Kliegel et al., 2013) and there is evidence that WM capacity, goal maintenance, inhibition and other related cognitive abilities increase from the age of six (Marcovitch et al., 2007; Towse et al., 2007; Marcovitch et al., 2010; Henry, 2011; López-Vicente et al., 2016). First, in line Kliegel et al. (2013) findings, we expected to observe better PM performance in the older group than in the younger group in the non-focal condition, but no age differences in the focal condition. Second, based on the findings of Smith et al. (2010), we expected the non-focal PM task to produce worse OT performance in both age groups relative to the focal condition. Smith et al. (2010) observed a PM cost in 6- and 10-year-old children when a single-task condition was compared to another condition including a non-focal PM task. Regarding the focal condition, and based on Leigh and Marcovitch (2014) results, we expected to observe a PM cost in our younger (6-year-old) group. Our expectations for the 10-year-old group were less clear, since no study has yet reported data on focal PM costs in late childhood. However, because overall age-related differences are usually more pronounced under non-focal cues (e.g., Rendell et al., 2007; Kliegel et al., 2008; Scullin et al., 2010; Wang et al., 2011), one could predict a lesser cost (or even no cost at all) in 11-year-old children (relative to 6-year-old children).

## Materials and Methods

### Participants

We recruited 95 children from a local primary school in Granada (Spain). The younger group consisted of 45 children (23 boys and 22 girls) who were 6 years old (*M* = 6.88, *SD* = 0.29), and the older group consisted of 50 children (26 boys and 24 girls) who were 11 years old (*M* = 11, *SD* = 0.39). The number of participants per group (approximately 50) was decided in advance based on the sample sizes considered in previous studies with children (e.g., Smith et al., 2010; Mahy et al., 2014). All participants were born in Spain and spoke Spanish as their mother language. The children were recruited through an informative talk for their parents in the school. The study was approved and carried out in accordance with the recommendations of the Research Ethics Committee of the University of Granada. All parents of participants were provided with information about the study and gave written informed consent in accordance with the Declaration of Helsinki. The participants belonged to families with medium socioeconomic status, as measured through their income index. To minimize the error variance, all participants performed all three experimental conditions: single-task, focal and non-focal. Hence, the study comprised a mixed design with age (6 vs. 11) and experimental condition as variables (between and within participants, respectively).

### Procedure

The experimental tasks used here were adapted from standard PM tasks used in previous studies with children (Mahy et al., 2014; Cottini et al., 2018). Before conducting the present experiment, a pilot study with ten 6-year-old children and ten 11-year-old children was carried out to ensure that children of these ages were able to successfully perform the focal and non-focal conditions and that we were able to obtain levels of performance similar to those of previous experiments. In the preliminary study, children were asked to perform the focal and non-focal tasks in random order. As the ongoing activity in both conditions, they had to categorize images that appeared on the screen as animals or not animals. In the focal condition, along with the categorization activity, children were asked not to categorize the ball or kite images but to press particular keys. In the non-focal condition, they had to stop the ongoing activity and press particular keys whenever the border of the screen changed to magenta or gray. The results of the pilot study showed that all the children were able to perform the PM task with levels of performance similar to previous studies (see Kliegel et al., 2013). In the focal condition, there was no difference between 6-year-old (*M =* 0.91, *SD =* 0.13) and 11-year-old (*M =* 0.92, *SD =* 0.06) children, *t*(18) < 1. In contrast, the analyses revealed statistically significant differences between the younger (*M =* 0.44, *SD =* 0.13) and the older (*M =* 0.71, *SD =* 0.09) children in the non-focal condition, *t*(18) = 5.23, *p* < 0.01, *d* = 2.41. Since these results are in line with those obtained by Kliegel et al. (2013), we conducted the proper experiment with a focus on the cost of focal and non-focal PM cues over the ongoing activity.

As in the preliminary study, testing was conducted individually in the school and lasted approximately 20 min. The testing session took place during school hours, and the children were taken out of their classroom during the testing. Each session consisted of three parts corresponding to each of the experimental conditions (single-task, focal and non-focal), whose order of administration was randomized.

In all three conditions, children were asked to perform a single task (OT) that consisted of categorizing pictures as animal or non-animal. We used 65 images taken from the work of Rossion and Pourtois (2004). Each was repeated twice during the three parts of the experiment. Half of the images referred to animals, and the other half did not. The stimuli appeared in the center of the screen surrounded by 15 pixel color border, which was randomly changed for each presentation of the stimuli (red, blue, green or yellow). The children were asked to press the key “yes” (placed on the “a” in the keyboard) whenever an animal item appeared and the key “no” (placed on the “s) whenever a non-animal item appeared. In the focal condition, the prospective focal task was included in the OT. Children were asked to remember to press a different key whenever a target picture (a kite or a ball) appeared. Whenever the kite appeared, they had to press the key “start” (placed on the “k”), and whenever the ball appeared, they had to press the key “square“ (placed on the “l“). In the non-focal condition, the children performed the OT and were also asked to press a different key when the picture frame had a particular color (magenta or gray). Specifically, whenever the screen border was magenta, they were asked to press the key “start (placed on the “k“), and whenever the border was gray, they were to press the key “square” (place on the “l”).

In each condition, the experiment had the following structure: First, the participants received the instructions for the single-task condition and practiced the task on nine trials. Then, after being informed that the tests had started, they moved to the experimental trials. The order in which the three conditions were presented to each participant was random. In the single-task condition, the participants faced 50 trials. In the focal condition, they were told about the prospective intention and practiced the OT task, which included four PM targets. When they correctly performed two of these four targets, they started the test that included 50 ongoing trials with five PM trials. We chose this PM trial frequency based on previous studies with children of similar ages (Kliegel and Jäger, 2007; Ford et al., 2012; ). The non-focal condition used the same structure, but involved instructing the participants about the non-focal cues. There was a short break (about 2 min.) between conditions, during which the children were given the instructions to perform the next block: “Now, I am going to explain the next game to you. Are you ready for this?” After all participants had been assessed, they received a gift for their participation.

The PM trials appeared in the focal condition in the 10th, 23rd, 32nd, 42nd and 54th positions. In the non-focal condition, the PM trials were in the 8th, 19th, 32nd, 45th and 54th positions. In the focal condition, two of the PM trials showed a kite, two showed a ball, and the fifth cue varied randomly across participants. In the non-focal condition, half of the target’s frames were magenta, and the rest were grey. The dependent measures were the proportion of correct responses and the reaction time.

Stimuli presentation during the OT and PM trials was set to a minimum of 1600 ms and a maximum of 2800 ms. When participants responded after 1600 ms, the next trials occurred after an inter-stimulus interval (ISI) of 250 ms. A response latency shorter than 1600 ms was filled with a black screen until 1600 ms, followed by the inter stimulus interval. If the participant did not respond within 2800 ms, the inter-stimulus interval appeared.

## Results

While the focus of the present experiment is on the performance of an ongoing task, we also report analyses of PM performance for the sake of completeness. As expected from the pilot study [and also the study by Kliegel et al. (2013)], focality had a reliable effect for the 6-year-old group [*F*(1,44) = 16.18, *MSe =* 0.01, *p* < 0.01, $\eta_{p}^{2}$ = 0.27], indicating better performance when the cue was focal (*M* = 0.74, *SD* = 0.32) relative to the condition in which the cue was non-focal (*M* = 0.50, *SD* = 0.32). The same pattern was observed in the 11-year-old group [*M* = 0.92, *SD* = 0.16 vs. *M* = 0.82, *SD* = 0.21; *F*(1,49) = 52.07, *MSe* = 0.00, *p* < 0.01, $\eta_{p}^{2}$ = 0.51].

Performance in the ongoing task was analysed first by conducting a 3 (condition) by 2 (age) ANOVA on the proportion of correct responses (Table 1). For each participant, correct responses were averaged across conditions and introduced into the analysis. Errors were evenly distributed across stimuli and participants with no outliers. The analysis of accuracy showed an effect of age [*F*(1,93) = 26.24, *MSe =* 0.01, *p* < 0.01, $\eta_{p}^{2}$ = 0.22], such that the older group performed the OT better than the younger group. In addition, there was a reliable effect of condition [*F*(2,92) = 41.42, *MSe =* 0.01, *p* < 0.01, $\eta_{p}^{2}$ = 0.31]. *Post-hoc* analyses using Bonferroni tests indicated that performance was reliably lower in the non-focal condition than in the focal and single-task conditions.

**Table 1:** Means of the proportions of correct OT responses and reaction times (in ms).

	6 years (*n* = 45)		11 years(*n* = 50)		Mean	
			
	*ACC*	*RT*	*ACC*	*RT*	*ACC*	*RT*
Single	0.93(0.07)	1213(178)	0.97(0.04)	939(135)	0.95(0.06)	1076(156)
Focal	0.89(0.12)	1322(150)	0.97(0.04)	1018(133)	0.93(0.08)	1170(141)
Non-focal	0.81(0.14)	1498(207)	0.91(0.07)	1331(135)	0.86(0.11)	1414(171)
Mean	0.88(0.11)	1344(178)	0.95(0.05)	5096(134)		

*Standard deviations are reported in brackets.*

**FIGURE 1:**
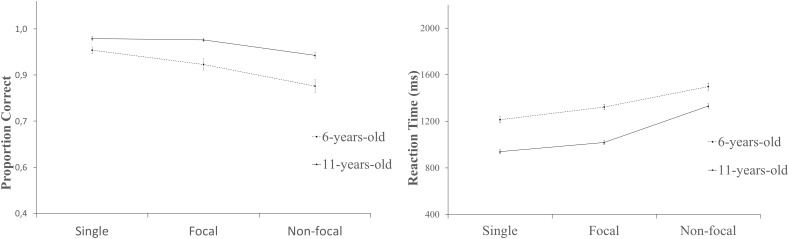
Ongoing performance. **(A)** Proportion of correct responses as a function of age and condition. **(B)** Reaction time as a function of age and condition. Error bars represent standard desviations.

More relevant, there was a reliable interaction between age and task condition [see Figure 1; *F*(2,92) = 4.59 *MSe =* 0.01, *p* = 0.01, $\eta_{p}^{2}$ = 0.05], which was followed up by analyzing the effects of condition on each age group. The analysis revealed a statistically significant effect in the 6-year-old group [*F*(2,43) = 20.18, *MSe =* 0.01, *p* < 0.01 $\eta_{p}^{2}$ = 0.31]. Further analyses indicated that performance was reliably worse in the non-focal condition than in the focal [*t*(44) = 3.30, *p* < 0.01, *d* = 0.53] and single-task [*t*(44) = 6.57, *p* < 0.01, *d* = 1.05] conditions. The difference between the focal and single-task conditions also reached statistical significance [*t*(44) = 2.89, *p* < 0.01, *d* = 0.47].

There was also a reliable effect of condition in the older group of children [*F*(2,48) = 27.85, *MSe =* 0.00, *p* < 0.01, $\eta_{p}^{2}$ = 0.36]. Performance in the non-focal condition differed from performance in the focal [*t*(49) = 5.69, *p* < 0.01, *d* = 0.91] and single-task conditions [*t*(49) = 6.33, *p* < 0.01, *d* = 0.95]. In this group of children, however, the performance difference between the single and focal conditions was not reliable [*t*(49) < 1, *d* = –0.10].

We also performed a 3 (condition) by 2 (age) ANOVA for reaction times on the ongoing task. Results of this analysis revealed a reliable effect of condition [*F* (2,92) = 263.37, *MSe =* 11555.34*, p* < 0.01, $\eta_{p}^{2}$ = 0.74]. Bonferroni tests indicated that responses were slower in the non-focal condition than the focal and single-task conditions. Reaction times in the single-task condition also differed from those in focal condition. The age effect was also reliable [*F*(1,93) = 82.97, *MSe =* 52787.49, *p* < 0.01, $\eta_{p}^{2}$ = 0.47]. The younger group took longer to respond than the older group (*M* = 1096.53, *SD* = 134.86). More importantly, there was a reliable interaction [*F*(2,92) = 11.29, *MSe =* 11555.34, *p* < 0.01, $\eta_{p}^{2}$ = 0.11; see Figure 1]. The one-way ANOVA in the 6-year-old group showed the effect of condition to be reliable [*F*(2,43) = 64.11, *MSe =* 16512.18, *p* < 0.01, $\eta_{p}^{2}$ = 0.59]. Reaction times were longer for non-focal trials than for focal [*t*(44) = 7.37, *p* < 0.01, *d* = 0.97] and single [*t*(44) = 9.59, *p* < 0.01, *d* = 1.57] trials. These children were also slower at responding in the focal than the single-task condition [*t*(44) = 4.97, *p* < 0.01, *d* = 0.66). A similar pattern of results emerged in the 11-year-old group [*F*(2,48) = 24.92, *MSe =* 7824.79, *p* < 0.01, $\eta_{p}^{2}$ = 0.85]. This group was slower when responding to the OT in the non-focal condition than in the focal [*t*(49) = 17.29, *p* < 0.01, *d* = 2.33] and single-task [*t*(49) = 22.3, *p* < 0.01, *d* = 2.88 conditions. Reaction times for the single trials were faster than reaction times for the focal trials [*t*(49) = 4.52, *p* < 0.01, *d* = 0.58].

## Discussion

The purpose of the present experiment was two fold: First, we aimed to test some of the predictions of the dual process framework (McDaniel et al., 2015) by comparing young children’s performance on the OT when cue focality was varied. The idea was that performance on the OT would provide an index of the cognitive costs associated with holding a prospective intention and monitoring for appropriate cues. Our second aim was to assess early developmental changes in the effect of the intention over the OT. Previous research with adult participants (Smith, 2003) has shown a PM cost over the OT when a PM intention is included. This cost has also been observed in 4-, 5- and 6-year-old children (Leigh and Marcovitch, 2014). To estimate this cost, we compared performance in a single-task condition (in which children only performed the OT) with performance in two prospective memory conditions varying in cue focality (focal vs. non-focal).

The results for the OT show an interesting pattern. On one hand, the reaction time findings are partly in line with the predictions of the PAM theory: For both younger and older children, holding a PM intention produced an OT cost such that they were faster in the single-task condition than in the focal and non-focal conditions. According to the PAM theory, to retrieve an intention and perform the action, an individual must maintain a state of readiness and monitor the elements of both the OT and the environment for PM cues (Smith, 2003). Interestingly, the cost varied across conditions, such that non-focal cues produced longer reaction times, which suggests that different cue conditions require different degrees of attention. Similarly, the reaction time findings showed that holding the intention was more costly for younger than for older children and that the difference between focal and non-focal cues was also more pronounced for younger children. On the other hand, the accuracy during the OT showed a very similar pattern so that younger and older children exhibited different degrees of impairment depending on the focality of the cue. Thus, although both had a similar cost when the cues were non-focal, older children were more efficient in the focal condition. Despite the focal and single-task conditions not showing differences in accuracy, the differences in reaction time between these two conditions suggest that both focal and non-focal PM tasks produce a cost over the OT, even though this cost is less pronounced in the focal task and in older children. These findings agree, in part, with the dual process framework (McDaniel et al., 2015) and suggest that when there is an overlap between the processing required to perform the OT and the prospective task, remembering the intention is less effortful and that different degrees of attention are involved in processing focal and non-focal cues.

Our pattern of results was similar to that observed by Wang et al. (2011), who found that adolescents and adults exhibited greater cost over the OT when the cue was non-focal than when it was focal. Hence, our older children behaved as adolescents and young adults usually do. While our study did not include any measure of executive functioning, previous studies have related some executive control processes, such as flexibility (Mahy and Munakata, 2015), inhibitory control (Wang et al., 2008) and monitoring abilities (Nigro et al., 2014), to PM performance. Thus, our finding that the older children outperformed the younger ones could be related to the development of their executive functioning. Future studies using the present experimental paradigm should include measures of executive function.

From a developmental perspective, our findings suggest that there are relevant changes from 6 to 11 years of age that make older children more efficient than their younger counterparts in dealing with PM tasks. Older children committed fewer errors and had shorter reaction times than younger children when they performed the OT while trying to remember a PM intention. Interestingly, the interaction between focality and age for OT response times showed that the older children were better able to reallocate their attention depending on the difficulty of the task. Thus, the magnitude of the difference between the focal and non-focal conditions was larger (Cohen’s *d* = 2.33) for the older group than the younger group (Cohen’s *d* = 0.97). Although both increased their times when the PM cue was non-focal (the difference between focal and non-focal conditions was significant for both groups), this increment was larger for the older children. Hence, the older children seemed better able to detect the difficulty of the non-focal task and allocate more resources (relative to the younger children) to cue monitoring. In line with this finding, previous studies have shown that metacognition affects PM performance. Kvavilashvili and Ford (2014), for example, found that PM performance was more accurate in children who better predicted their own performance. More recently, Cottini et al. (2018) found better performance in a categorical PM task in children with high declarative metamemory (relative to children with low metamemory). Importantly, metamemory was found to have no effect on the specific PM task, which is thought to be less demanding than the categorical task. These findings support the theory that children who are good at predicting how well they will do on PM tasks are also better able to choose the most appropriate strategy to deal with the task at hand. Hence, performance from our older children could have stemmed from better predictions of their PM performance, which, in turn, allowed for better adjustment to the requirements of the focal and non-focal tasks. While this interpretation fits well with our results, it should be corroborated in future studies involving more direct measures.

However, the fact that both 6- and 11-year-old children performed better on focal than non-focal PM tasks suggests that, though less efficiently, younger children are also able to monitor for cues and be sensitive to their focality. These developmental findings are consistent with those of Smith et al. (2010), who showed a PM cost for 7- and 10-year-old children and adults, and those of Leigh and Marcovitch (2014), who found that this cost is also present in 4-year-old children. These findings suggest that even at a very young age, children can engage in preparatory attentional processes and monitoring strategies for a PM cue, thus reducing their performance on the OT.

Although the pattern of results is clear and consistent with previous findings, the study is not without limitations. First, although our sample size was large enough to detect the interaction between focality and age, a larger sample might have shown more pronounced age differences. Second, we only used one type of focal and non-focal cue, and it could be possible that other cues might have produced different results. For example, PM tasks involving less salient cues might produce greater cost in the children’s performance, with younger children showing more difficulties relocating the resources needed for remembering the intention. Hence, to be able to generalize our findings, further studies should include more than one type of PM task with different relations between the PM and the OT (e.g., a PM focal task in which the PM cue is not part of the materials used in the OT or a non-focal task involving other than perceptual information). Despite these limitations, our results are important and possess clear implications. Parents and teachers occasionally assume that when children start school they are prepared to effectively fulfil the responsibilities that are required at school, such as giving their parents a permission slip or remembering to bring course materials. However, our results suggest that these tasks can be highly demanding for them and that younger children might need to learn simple strategies that help them efficiently allocate their resources to be able to recall their intentions.

In sum, the results of the present study show that, under some conditions (focal cues and older children), holding a PM intention produces lower costs during OT performance and that, therefore, cue monitoring and intentional retrieval might not always play a main role in PM. This evidence is partially in line with the dual process framework (McDaniel et al., 2015), since the fact that reaction times were slower for both the focal and the non-focal conditions relative to the single-task conditions for both younger and older children suggests that the involvement of attentional processes is a question of degree, such that either more or less resources are necessary depending on task focality. While focal PM tasks affect RT but not accuracy, non-focal tasks hamper both accuracy and response times. This pattern advances an interesting problem, given that the role of cue focality is not completely clear; while some studies have failed to reveal differences between focal and non-focal cues (Kliegel et al., 2013), others have shown the opposite pattern: namely, a greater cost for focal than non-focal cues (Ball and Bugg, 2018). Most likely, cue focality interacts with the type of OT, such that effective resource allocation to the PM task depends on the amount of demands of both the PM and the OT. Further studies should explore this interaction and how it modulates children’s ability to strategically remember performing actions in the future.

## Author Contributions

This work is part of the thesis dissertation of the first author (AC). AC and MB developed the idea for the study together. AC contributed to the design of the PM tasks, the data collection and analyses, and the manuscript writing. MB and CG-A supervised the study and the analyses, and wrote the manuscript, and reviewed and approved the final version of the manuscript.

## Conflict of Interest Statement

The authors declare that the research was conducted in the absence of any commercial or financial relationships that could be construed as a potential conflict of interest.

## References

[B1] Anderson N. D. Craik F. I. Naveh-Benjamin M. (1998). The attentional demands of encoding and retrieval in younger and older adults: 1. evidence from divided attention costs. *Psychol. Aging* 13 405–423. 10.1037/0882-7974.13.3.405 9793117

[B2] Ball B. H. Bugg J. M. (2018). Context cue focality influences strategic prospective memory monitoring. *Psychon. Bull. Rev.* 25 1405–1415. 10.3758/s13423-018-1442-9 29435962 PMC6070398

[B3] Basso D. Ferrari M. Palladino P. (2010). Prospective memory and working memory: asymmetrical effects during frontal lobe TMS stimulation. *Neuropsychologia* 48 3282–3290. 10.1016/j.neuropsychologia.2010.07.011 20637788

[B4] Brandimonte M. A. Einstein G. O. McDaniel M. A. (1996). *Prospective Memory: Theory and Applications.* Mahwah, NJ: Erlbaum.

[B5] Cheie L. Macleod C. Miclea M. Visu-Petra L. (2017). When children forget to remember: effects of reduced working memory availability on prospective memory performance. *Mem. Cognit.* 45 651–663. 10.3758/s13421-016-0682-z 27987114

[B6] Cottini M. Basso D. Palladino P. (2018). The role of declarative and procedural metamemory in event-based prospective memory in school-aged children. *J. Exp. Child Psychol.* 166 17–33. 10.1016/j.jecp.2017.08.002 28858667

[B7] Craik F. I. Govoni R. Naveh-Benjamin Ben-Gurion M. Anderson N. D. Cowan N. Johnston W. (1996). The effects of divided attention on encoding and retrieval processes in human memory. *J. Exp. Psychol.* 125 159–180. 10.1037/0096-3445.125.2.159 8683192

[B8] Einstein G. O. Mcdaniel M. A. (2005). Prospective memory multiple retrieval processes. *Curr. Dir. Psychol. Sci.* 14 286–290. 10.1111/j.0963-7214.2005.00382.x

[B9] Einstein G. O. McDaniel M. A. Manzi M. Cochran B. Baker M. (2000). Prospective memory and aging: forgetting intentions over short delays. *Psychol. Aging* 15 671–683. 10.1037//0882-7974.15.4.671 11144326

[B10] Einstein G. O. Smith R. E. Mcdaniel M. A. Shaw P. (1997). Aging and prospective memory: the influence of increased task demands at encoding and retrieval. *Psychol. Aging* 12 479–488. 10.1037/0882-7974.12.3.479 9308095

[B11] Ford R. M. Driscoll T. Shum D. Macaulay C. E. (2012). Executive and theory-of-mind contributions to event-based prospective memory in children: exploring the self-projection hypothesis. *J. Exp. Child Psychol.* 111 468–489. 10.1016/j.jecp.2011.10.006 22169353

[B12] Harrison T. L. Einstein G. O. (2010). Prospective memory: are preparatory attentional processes necessary for a single focal cue? *Mem. Cognit.* 38 860–867. 10.3758/MC.38.7.860 20921099

[B13] Henry J. D. Macleod M. S. Phillips L. H. Crawford J. R. (2004). A meta-analytic review of prospective memory and aging. *Psychol. Aging* 19 27–39. 10.1037/0882-7974.19.1.27 15065929

[B14] Henry L. (2011). *The Development of Working Memory in Children.* Newcastle upon Tyne: Sage.

[B15] Kliegel M. Jäger T. (2007). The effects of age and cue-action reminders on event-based prospective memory performance in preschoolers. *Cogn. Dev.* 22 33–46. 10.1016/j.cogdev.2006.08.003

[B16] Kliegel M. Jäger T. Phillips L. H. (2008). Adult age differences in event-based prospective memory: a meta-analysis on the role of focal versus nonfocal cues. *Psychol. Aging* 23 203–208. 10.1037/0882-7974.23.1.203 18361667

[B17] Kliegel M. Mahy C. E. V. Voigt B. Henry J. D. Rendell P. G. Aberle I. (2013). The development of prospective memory in young schoolchildren: the impact of ongoing task absorption, cue salience, and cue centrality. *J. Exp. Child Psychol.* 116 792–810. 10.1016/j.jecp.2013.07.012 24056203

[B18] Kliegel M. Martin M. McDaniel M. A. Einstein G. O. (2002). Complex prospective memory and executive control of working memory: a process model. *Psychol. Database* 44 303–318.

[B19] Knight J. B. Meeks J. T. Marsh R. L. Cook G. I. Brewer G. A. Hicks J. L. (2011). Recall and recognition of dreams and waking events: a diary paradigm. *Learn. Mem. Cogn.* 37 298–307. 10.1037/a0021969 21299328

[B20] Kvavilashvili L. Ford R. M. (2014). Metamemory prediction accuracy for simple prospective and retrospective memory tasks in 5-year-old children. *J. Exp. Child Psychol.* 127 65–81. 10.1016/j.jecp.2014.01.014 24698432

[B21] Kvavilashvili L. KyLe F. Messer D. J. (2008). “The development of prospective memory in children: Methodological issues, empirical findings, and future direction,” in *Prospective Memory: Cognitive, Neuroscience, Developmental, and Applied Perspectives*, eds Kliegel M. McDaniel M. A. Einstein G. O. (Mahwah NJ: Lawrence Erlbaum Associates), 115–140.

[B22] Kvavilashvili L. Messer D. J. Ebdon P. (2001). Prospective memory in children: the effects of age and task interruption. *Dev. Psychol.* 37 418–430. 10.1037/0012-1649.37.3.418 11370916

[B23] Leigh J. Marcovitch S. (2014). The cognitive cost of event-based prospective memory in children. *J. Exp. Child Psychol.* 127 24–35. 10.1016/j.jecp.2014.02.010 24853249

[B24] López-Vicente M. Forns J. Suades-González E. Esnaola M. García-Esteban R. Álvarez-Pedrerol M. (2016). Developmental trajectories in primary schoolchildren using n-back task. *Front. Psychol.* 7:716. 10.3389/fpsyg.2016.00716 27242625 PMC4866535

[B25] Mahy C. E. V. Moses L. J. (2011). Executive functioning and prospective memory in young children. *Cogn. Dev.* 26 269–281. 10.1016/j.cogdev.2011.06.002

[B26] Mahy C. E. V. Moses L. J. Kliegel M. (2014). The impact of age, ongoing task difficulty, and cue salience on preschoolers’ prospective memory performance: the role of executive function. *J. Exp. Child Psychol.* 127 52–64. 10.1016/j.jecp.2014.01.006 24613075

[B27] Mahy C. E. V. Munakata Y. (2015). Transitions in executive function: insights from developmental parallels between prospective memory and cognitive flexibility. *Child Dev. Perspect.* 9 128–132. 10.1111/cdep.12121

[B28] Marcovitch S. Boseovski J. J. Knapp R. J. (2007). REPORT Use it or lose it: examining preschoolers’ difficulty in maintaining and executing a goal. *Dev. Sci.* 10 559–564. 10.1111/j.1467-7687.2007.00611.x 17683342

[B29] Marcovitch S. Boseovski J. J. Knapp R. J. Kane M. J. (2010). Goal neglect and working memory capacity in 4-to 6-year-old children. *Child Dev.* 81 1687–1695. 10.1111/j.1467-8624.2010.01503.x 21077857

[B30] McDaniel M. A. Einstein G. O. (2000). Strategic and automatic processes in prospective memory retrieval: a multiprocess framework. *Appl. Cogn. Psychol.* 14 S127–S144. 10.1002/acp.775

[B31] McDaniel M. A. Umanath S. Einstein G. O. Waldum E. R. (2015). Dual pathways to prospective remembering. *Front. Hum. Neurosci.* 9:392. 10.3389/fnhum.2015.00392 26236213 PMC4500919

[B32] Nigro G. Brandimonte M. A. Cicogna P. C. Cosenza M. (2014). Episodic future thinking as a predictor of children’s prospective memory. *J. Exp. Child Psychol.* 127 82–94. 10.1016/j.jecp.2013.10.013 24332788

[B33] Park D. C. Hertzog C. Kidder D. P. Morrell R. W. Mayhorn C. B. (1997). Effect of age on event-based and time-based prospective memory. *Psychol. Aging* 12 314–327. 10.1037/0882-7974.12.2.314 9189992

[B34] Rendell P. G. McDaniel M. A. Forbes R. D. Einstein G. O. (2007). Age-related effects in prospective memory are modulated by ongoing task complexity and relation to target cue aging and prospective memory. *Neuropsychol. Cogn. Aging* 14 236–256. 10.1080/13825580600579186 17453559

[B35] Rossion B. Pourtois G. (2004). Revisiting snodgrass and vanderwart’s object pictorial set: the role of surface detail in basic-level object recognition. *Perception* 33 217–236. 10.1068/p5117 15109163

[B36] Scullin M. K. McDaniel M. A. Einstein G. O. (2010). Control of cost in prospective memory: evidence for spontaneous retrieval processes. *J. Exp. Psychol. Learn. Mem. Cogn.* 36 190–203. 10.1037/a0017732 20053054

[B37] Scullin M. K. Mcdaniel M. A. Shelton J. T. Lee J. H. Scullin M. K. Mcdaniel M. A. (2011). Focal/Nonfocal cue effects in prospective memory?: monitoring difficulty or different retrieval processes. 36 736–749. 10.1037/a0018971.Focal/Nonfocal PMC2864946 20438269

[B38] Smith R. E. (2003). The cost of remembering to remember in event-based prospective memory: investigating the capacity demands of delayed intention performance. *J. Exp. Psychol. Learn. Mem. Cogn.* 29 347–361. 10.1037/0278-7393.29.3.347 12776746

[B39] Smith R. E. Bayen U. J. (2005). The effects of working memory resource availability on prospective memory. *Exp. Psychol.* 52 243–256. 10.1027/1618-3169.52.4.243 16304724

[B40] Smith R. E. Bayen U. J. Martin C. (2010). The cognitive processes underlying event-based prospective memory in school-age children and young adults: a formal model-based study. *Dev. Psychol.* 46 230–244. 10.1037/a0017100 20053020 PMC2856082

[B41] Smith R. E. Hunt R. R. Mcvay J. C. Mcconnell M. D. (2007). The cost of event-based prospective memory: salient target events. *J. Exp. Psychol. Learn. Mem. Cogn.* 33 734–746. 10.1037/0278-7393.33.4.734 17576150

[B42] Towse J. N. Lewis C. Knowles M. (2007). When knowledge is not enough: the phenomenon of goal neglect in preschool children. *J. Exp. Child Psychol.* 96 320–332. 10.1016/j.jecp.2006.12.007 17300798

[B43] Wang L. Kliegel M. Liu W. Yang Z. (2008). Prospective memory performance in preschoolers: inhibitory control matters. *Eur. J. Dev. Psychol.* 5 289–302. 10.1080/17405620600778161

[B44] Wang L. Liu W. Xiong W. Akgün C. Kliegel M. (2011). Prospective memory across adolescence: the effects of age and cue focality. *Dev. Psychol.* 47 226–232. 10.1037/a0021306 21244161

[B45] Zimmermann T. D. Meier B. (2006). The rise and decline of prospective memory performance across the lifespan. *Q. J. Exp. Psychol.* 59 2040–2046. 10.1080/17470210600917835 17095485

[B46] Zöllig J. West R. Martin M. Altgassen M. Lemke U. Kliegel M. (2007). Neural correlates of prospective memory across the lifespan. *Neuropsychologia* 45 3299–3314. 10.1016/j.neuropsychologia.2007.06.010 17675111

